# Intra- and inter-rater reliability of rectus femoris muscle thickness measured using ultrasonography in healthy individuals

**DOI:** 10.1186/s13089-021-00224-8

**Published:** 2021-04-15

**Authors:** Yosuke Takahashi, Yuji Fujino, Kohei Miura, Ayumi Toida, Tadamitsu Matsuda, Shigeru Makita

**Affiliations:** 1 Department of Rehabilitation Center, Saitama Medical University International Medical Center, 1397-1 Yamane, Hidaka, Saitama 350-1298 Japan; 2 Department of Physical Therapy, Faculty of Health Science, Juntendo University, 3-2-12, Hongo, Bunkyo-ku, Tokyo 113-0033 Japan; 3 Department of Central Examination, Saitama Medical University International Medical Center, 1397-1 Yamane, Hidaka, Saitama 350-1298 Japan; 4 Department of Cardiac Rehabilitation, Saitama Medical University International Medical Center, Hidaka, Saitama 1397-1 Yamane350-1298 Japan

**Keywords:** Skeletal muscle mass, Reliability, Ultrasonography, Rectus femoris muscle

## Abstract

**Background:**

Ultrasonography (US) is a feasible and accessible method for the measurement of skeletal muscle mass. This technique presents acceptable intra-rater reliability; however, there are a few reports on its inter-rater reliability. Additionally, relative reliability should equally be inspected to determine the presence of systematic errors. Therefore, this study aimed to investigate the intra- and inter-rater reliabilities and absolute reliability of rectus femoris muscle thickness as measured using US.

**Methods:**

The participants included in our study comprised 12 healthy young men (26.5 ± 3.9 years. Rectus femoris muscle thickness was measured from the right side of the thigh using US by two trained physical therapists. Inter- and intra-rater reliabilities were determined using the intraclass correlation coefficient (ICC) (1, 1) and ICC (2, 1) methods, respectively. Absolute reliability was evaluated using Bland − Altman analysis. Additionally, we calculated the minimal detectable change at the 95% level of confidence (MDC_95_).

**Result:**

According to the results of the Bland − Altman analysis, no fixed or proportional errors were present. The ICC (1, 1) was 0.95, and the ICC (2, 1) was 0.70. The MDC_95_ values of rectus femoris thickness for the intra- and inter-rater reliabilities were 2.0 mm and 4.3 mm, respectively.

**Conclusions:**

In our study, intra- and inter-rater reliabilities were measured at “excellent” and “moderate” levels in the healthy individuals based on a previously defined scale. Moreover, we determined the measurement error for quantifying rectus femoris thickness. Therefore, the measurement of rectus femoris thickness using US could be considered applicable in clinical research.

## Background

Loss of skeletal muscle mass is a major and well-known quantitative change associated with aging. Similarly, qualitative changes in skeletal muscle have been reported to correlate with aging, such as the preferential atrophy of type II muscle fibers [1, 2], increased intramuscular fat [3–5], and increased extracellular water volume relative to muscle volume [6]. The gold standard methods for skeletal muscle mass measurement are computed tomography (CT), magnetic resonance imaging (MRI), and dual-energy X-ray absorptiometry (DEXA) [7]. However, these techniques are not optimal for measuring qualitative changes in the skeletal muscle. Furthermore, they are not frequently used as they are costly and infer a risk of radiation exposure in a clinical setting. Ultrasonography (US) measurement is a more feasible and accessible method that can quantitatively and qualitatively evaluate muscles. Muscle thickness measured using US is similar to that measured using CT, MRI, or DEXA [8–11]. Therefore, US measurement has rapidly spread in the field of physical therapy.

The measurement of muscle thickness using US presents acceptable intra-rater reliability; however, there are a few reports on its inter-rater reliability [12–14]. Moreover, relative reliability cannot distinguish the type and extent of errors included in the measured values, as this statistical method is based on the assumption that only random errors occur. The measured error can be either systematic or random, and it is difficult to overcome systematic errors through repeated measurements. Hence, it is necessary to determine the presence of systematic errors.

## Materials and methods

### Aim and study design

This study aimed to investigate the intra- and inter-rater reliabilities and absolute reliability of measuring rectus femoris muscle thickness using US. The present study design was a test–retest study (within-day and 1 week apart).

### Participants

A poster for research recruitment was posted in the first author's hospital to recruit participants for this study. Over a 12-month period (March 2017 to March 2018), 12 young male volunteers with a mean age of 26.5 ± 3.9 years (mean ± standard deviation) participated in this study. Participant characteristics are shown in Table 1. The inclusion criteria included: (1) over 20 years old; (2) under 40 years old; (3) no orthopedic problem; (4) not hypersensitive to electrical stimulation; (5) able to provide informed consent. All participants received an explanation of the study purpose and provided written informed consent. The study was approved by the ethics committees of Saitama Medical University International Medical Center (16–188).

**Table 1:** Demographics of the participants

Characteristic	Total (*n* = 12)
Age (years)	26.5 ± 3.9^a^
Height (cm)	174.3 ± 0.1^a^
Weight (kg)	64.9 ± 7.2^a^
BMI (kg/m^2^)	21.3 ± 1.9^a^

*BMI* body mass index

^a^Values are presented as mean ± standard deviation

### Measurement of rectus femoris muscle thickness

**Fig. 1:**
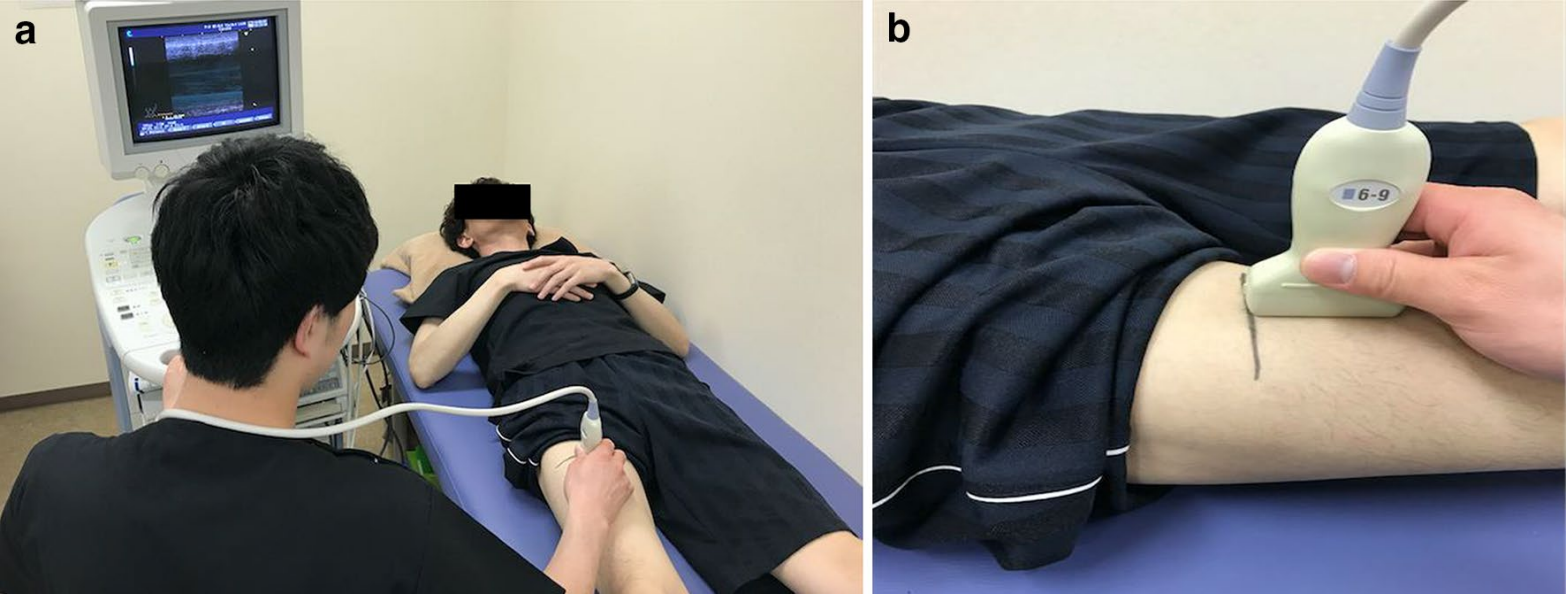
Ultrasonography set-up in the measurement of skeletal muscle thickness. **a** Participant is depicted in a supine position while the user holds the ultrasound probe against the anterior surface of the thigh. **b** The exact position of the probe is illustrated. Muscle thickness was determined from the resulting ultrasound images, and the values from two independent raters were used to decipher the intra- and inter-rater reliabilities

**Fig. 2:**
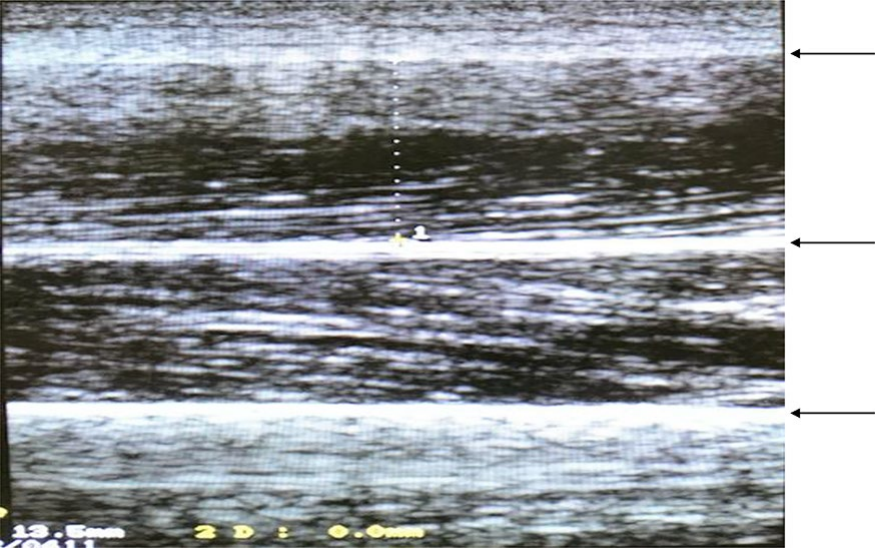
Ultrasound of rectus femoris muscle. Ultrasound images of the muscular layers were obtained, and the muscle thickness of the rectus femoris was measured by drawing a perpendicular line from the border lines of the rectus femoris to the border lines of the vastus intermedius. On the screen of the US (ultrasonography) device, muscle thickness was calculated by setting the cursor to two points from the border lines of the rectus femoris to the border lines of vastus intermedius. Top arrow: rectus femoris muscle; middle arrow: vastus intermedius muscle; bottom arrow: femur

**Fig. 3:**
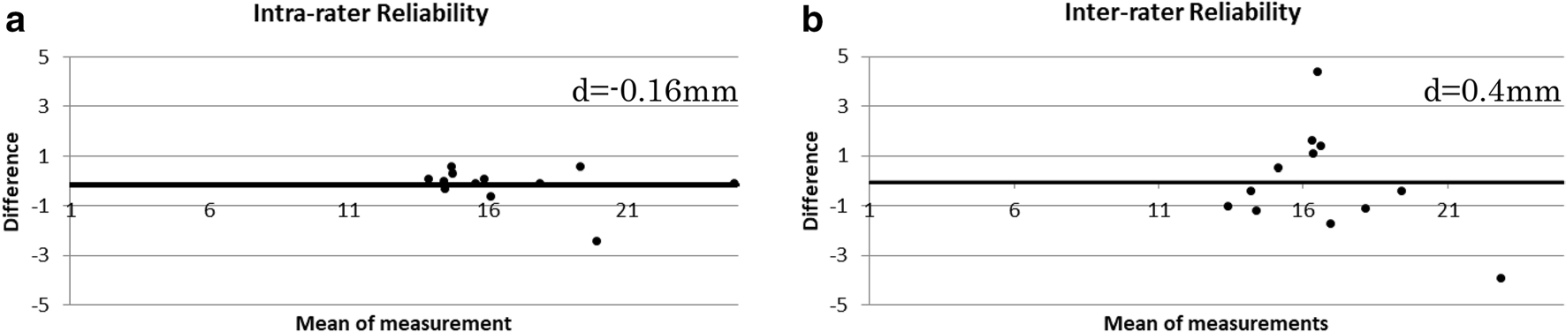
Bland–Altman plots of intra- and inter-rater reliabilities of ultrasonography to measure skeletal muscle thickness. The values from two independent raters were used to decipher the intra- and inter-rater reliabilities. Bland–Altman plots of the mean measurement against the difference between the measurements are depicted for intra-rater reliability (**a**) and inter-rater reliability (**b**)

In the measurements performed using US, a transducer probe (UF-850XTD: Fukuda Denshi Co. Ltd., Tokyo, Japan) was used, as illustrated in Fig. 1b. Participants lay in the supine position with the knee extended and maintained this posture throughout the US measurement (Fig. 1). Rectus femoris muscle thickness was measured from the right side of the thigh. The ultrasound probe was placed on the anterior surface of the thigh, at the midpoint of the length between the anterior superior iliac spine and the upper border of the patella, referring to the measurement position of Pardo et al. [14]. The measurement sites were determined using a measuring tape and marked with a permanent marker. A circumferential mark was made perpendicularly at the midpoint of the long axis. The probe was placed at the top of this circumferential line (Fig. 2) and moved along the line until a suitable image was obtained, in which a parallel position of the three borderlines of the rectus femoris, vastus intermedius, and femur was achieved (Fig. 3). On the screen of the US device, muscle thickness was calculated by setting the cursor to two points from the border lines of the rectus femoris to the borderlines of vastus intermedius. The probe was maintained in this perpendicular position on the skin surface, and minimal contact pressure was applied during the measurements to obtain good-quality images.

Rectus femoris muscle thickness was measured by two trained physical therapists (A and B). To investigate the intra-rater reliability, the measurement was performed twice with an interval of 1 week by only rater A. To investigate the inter-rater reliability, the measurement was concurrently performed by two independent raters (A and B); both raters were blinded to each other's results. We simultaneously focused on two or more measurements because we wanted to examine the inter-rater reliability by evaluating the concurrent measurement performed by two examiners.

### Data analysis

The intraclass correlation coefficient (ICC) with 95% confidence intervals (CI) was calculated to determine the relative reliability. Inter- and intra-rater reliabilities were determined using the ICC (1, 1) and ICC (2, 1) methods, respectively. The absolute reliability was evaluated using Bland − Altman analysis for obtaining intra- and inter-rater reliabilities. Furthermore, we calculated a confidence level of 95% of the average difference between the two measured values to detect the existence of fixed errors, and this was performed using the following formula:

$$\stackrel{-}{d}\pm t\times \sqrt{\frac{\mathrm{SD}d}{n},}$$

where $\stackrel{-}{d}$ is the average of 2 measurement values, *t* is the degree of freedom in the case of *n*−1, *n* is the sample size, and SD_d_ is the standard error of $\stackrel{-}{d}$

If the result did not contain 0, we concluded that a fixed error was present, assuming that the measured values were distributed in a fixed direction [15]. Further, we calculated the *T* value to inspect for the existence of a proportional error using the following formula:

$$t=r\sqrt{\frac{n-2}{1-{r}^{2}};}$$

*r* is calculated using the correl function in Excel.

If the calculated *T* value was higher than the *T* value when the degree of freedom was *n*−2, and the level of significance was 5%, we concluded that a proportional error was present, assuming that there was a significant correlation [15]. Additionally, we calculated the minimal detectable change at the 95% level of confidence [MDC_95_ = standard error of measurement (SEM) × 1.96 × √2], which is determined from the standard deviation of the difference (SDd): SEM = SDd × √2. This represents the smallest change that can be interpreted as a real difference using the following formula:

$${\text{MDC}}_{{95}} \; = \;1.96\; \times \;{\text{SDd}}$$

Statistical analysis was performed using PASW Statistics ver. 18.0 (SPSS Japan Inc, Tokyo, Japan), with the level of significance set at 5%.

## Results

The two measurements obtained by rater A were 16.7 ± 3.0 mm and 16.9 ± 3.3 mm, and the measurement obtained by rater B was 17.0 ± 2.3 mm (Table 2). According to the results of the Bland − Altman analysis, no fixed or proportional errors were present. The ICC (1, 1) for the intra-rater reliability was 0.95 (95% CI 0.856 − 0.987), and the ICC (2, 1) for the inter-rater reliability was 0.70 (95% CI 0.225−0.901). The MDC_95_ values of rectus femoris thickness for the intra-rater and inter-rater reliabilities were 2.0 mm and 4.3 mm, respectively (Table 3). The Bland−Altman plots were drawn (Fig. 4), and the differences in the scores were plotted against the mean scores of the measurements.

**Table 2:** Measurement values taken by 2 independent raters

No.	1st assessment	2nd assessment	Rater B (mm)
	Rater A (once) (mm)	Rater A (twice) (mm)	
1	15.6	15.4	18.1
2	14.5	14.4	19.5
3	18.8	21.6	17.7
4	17.6	18.4	16.3
5	14.9	15.3	16.2
6	15.5	14.4	14.0
7	14.4	14.4	14.3
8	19.5	18.9	20.0
9	14.5	14.5	15.4
10	24.3	24.5	20.5
11	14.3	14.8	14.0
12	16.8	16.3	17.5
Average	16.7	16.9	17.0
SD	3.0	3.3	2.3

*SD* standard deviation

**Table 3:** The relative and absolute reliability results

	Relative reliability	Absolute reliability				MDC_95_(mm)
	ICC (95% CI)	Fixed error		Proportional error		
		95% CI	Result	*T* value	result	
Intra-rater reliability	0.95 (0.856–0.987)	− 0.8 ~ 0.4	No	− 1.2	No	2.0
Inter-rater reliability	0.70 (0.257–0.901)	− 1.0 ~ 1.8	No	− 1.6	No	4.3

*ICC* intraclass correlation coefficient, *CI* confidence interval, *MDC95* minimal detectable change with 95% CI

## Discussion

In this study, we evaluated the intra- and inter-rater and absolute reliabilities of rectus femoris muscle measurement using US. Our findings support US as a useful tool for measuring rectus femoris muscle thickness in healthy young individuals. The primary use of analyses of healthy populations is to allow comparisons with pathological populations, and it follows, therefore, that measurement reliability in healthy populations is of importance. Few studies have compared MDC for intra- and inter-rater reliabilities; however, our study results indicated that rectus femoris muscle thickness should be measured by the same rater.

Based on the 95% CI Koo et al*.* [16] reported that ICC values less than 0.5, between 0.5 and 0.75, between 0.75 and 0.9, and greater than 0.9 indicate poor, moderate, good, and excellent reliability, respectively. The ICC values for the rectus femoris muscle thickness (intra-rater reliability = 0.99, inter-rater reliability = 0.96) presented here were similar to those reported by other studies [17, 18]. In this study, the ICCs of relative reliability was 0.95 for ICC (1,1) and 0.70 for ICC (2,1). ICC (1,1) and ICC (2,1), used to assess the intra- and inter-rater reliabilities, were at the “excellent” and “moderate” levels, respectively. Similarly, 95% CI was 0.856−0.987 for ICC (1,1) and 0.225−0.901 for ICC (2,1). ICC (1,1) had “good” to “excellent” reliability; however, ICC (2,1) significantly varied from “poor” to “excellent”. Therefore, the intra- rater reliability could be considered valid.

In addition, a systematic error was not detected in the absolute reliability results of the Bland−Altman analysis. The MDC_95_, a statistical parameter, can be used as a threshold to help users distinguish a real change from a measurement error for an individual participant. If the obtained value is equal to or less than the MDC_95_, then it is within the error range [19]. However, if the value is greater than the MDC_95_, it can be determined that a true change has occurred [19]. In this study, the MDC_95_ values for intra-rater and inter-rater reliabilities were found to be 2.0 mm and 4.3 mm, respectively, which is a useful index for judging the time course and therapeutic effect.

However, this study has some limitations. The amount of inward pressure on the patient caused by the transducer was not evaluated during the measurements. We believe that this is a possible reason why the inter-rater reliability was observed to be “moderate”. In other words, it is necessary to consider the difference in the rater’s measurement ability. These transducer conditions should have been precisely determined in the measurements obtained in the study. Another limitation is that only young males were enrolled in the study. Therefore, the influence of gender and age is unknown. Finally, a systematic error was not identified in this study. Thus, we should have calculated the appropriate repetition count to improve reliability. Further research is required to keep the amount of inward pressure caused by the transducer constant to ensure that inter-rater reliability is as good as intra-rater reliability. Similarly, more participants are needed to determine the effect of gender and age.

## Conclusions

In conclusion, the intra-rater reliability and inter-rater reliability of the measurement of rectus femoris muscle thickness using US were mostly sufficient in healthy young men. Moreover, we determined the measurement error for rectus femoris thickness. Measuring the MDC is useful when verifying the degree of muscle atrophy and any therapeutic effects via the strengthening of muscles. Therefore, the measurement of rectus femoris muscle thickness using US could be considered applicable in clinical research.

## Data Availability

The datasets used and/or analyzed during the current study are available from the corresponding author on reasonable request.

## Abbreviations

CT: Computed tomography
CI: Confidence interval
DEXA: Dual-energy X-ray absorptiometry
ICC: Intraclass correlation coefficient
MRI: Magnetic resonance imaging
MDC_95_: Minimal detectable change with 95% CI
SD: Standard deviation
SEM: Standard error of measurement
US: Ultrasonography
